# Measurement of the $\mathrm{t}\overline{\mathrm{t}}$ production cross section using events in the $\mathrm {e}\mu$ final state in pp collisions at $\sqrt{s}=13\,\text {TeV}$

**DOI:** 10.1140/epjc/s10052-017-4718-8

**Published:** 2017-03-20

**Authors:** V. Khachatryan, A. M. Sirunyan, A. Tumasyan, W. Adam, E. Asilar, T. Bergauer, J. Brandstetter, E. Brondolin, M. Dragicevic, J. Erö, M. Flechl, M. Friedl, R. Frühwirth, V. M. Ghete, C. Hartl, N. Hörmann, J. Hrubec, M. Jeitler, A. König, I. Krätschmer, D. Liko, T. Matsushita, I. Mikulec, D. Rabady, N. Rad, B. Rahbaran, H. Rohringer, J. Schieck, J. Strauss, W. Waltenberger, C.-E. Wulz, O. Dvornikov, V. Makarenko, V. Zykunov, V. Mossolov, N. Shumeiko, J. Suarez Gonzalez, S. Alderweireldt, E. A. De Wolf, X. Janssen, J. Lauwers, M. Van De Klundert, H. Van Haevermaet, P. Van Mechelen, N. Van Remortel, A. Van Spilbeeck, S. Abu Zeid, F. Blekman, J. D’Hondt, N. Daci, I. De Bruyn, K. Deroover, S. Lowette, S. Moortgat, L. Moreels, A. Olbrechts, Q. Python, S. Tavernier, W. Van Doninck, P. Van Mulders, I. Van Parijs, H. Brun, B. Clerbaux, G. De Lentdecker, H. Delannoy, G. Fasanella, L. Favart, R. Goldouzian, A. Grebenyuk, G. Karapostoli, T. Lenzi, A. Léonard, J. Luetic, T. Maerschalk, A. Marinov, A. Randle-conde, T. Seva, C. Vander Velde, P. Vanlaer, R. Yonamine, F. Zenoni, F. Zhang, A. Cimmino, T. Cornelis, D. Dobur, A. Fagot, G. Garcia, M. Gul, I. Khvastunov, D. Poyraz, S. Salva, R. Schöfbeck, A. Sharma, M. Tytgat, W. Van Driessche, E. Yazgan, N. Zaganidis, H. Bakhshiansohi, C. Beluffi, O. Bondu, S. Brochet, G. Bruno, A. Caudron, S. De Visscher, C. Delaere, M. Delcourt, B. Francois, A. Giammanco, A. Jafari, P. Jez, M. Komm, V. Lemaitre, A. Magitteri, A. Mertens, M. Musich, C. Nuttens, K. Piotrzkowski, L. Quertenmont, M. Selvaggi, M. Vidal Marono, S. Wertz, N. Beliy, W. L. Aldá Júnior, F. L. Alves, G. A. Alves, L. Brito, C. Hensel, A. Moraes, M. E. Pol, P. Rebello Teles, E. Belchior Batista Das Chagas, W. Carvalho, J. Chinellato, A. Custódio, E. M. Da Costa, G. G. Da Silveira, D. De Jesus Damiao, C. De Oliveira Martins, S. Fonseca De Souza, L. M. Huertas Guativa, H. Malbouisson, D. Matos Figueiredo, C. Mora Herrera, L. Mundim, H. Nogima, W. L. Prado Da Silva, A. Santoro, A. Sznajder, E. J. Tonelli Manganote, A. Vilela Pereira, S. Ahuja, C. A. Bernardes, S. Dogra, T. R. Fernandez Perez Tomei, E. M. Gregores, P. G. Mercadante, C. S. Moon, S. F. Novaes, Sandra S. Padula, D. Romero Abad, J. C. Ruiz Vargas, A. Aleksandrov, R. Hadjiiska, P. Iaydjiev, M. Rodozov, S. Stoykova, G. Sultanov, M. Vutova, A. Dimitrov, I. Glushkov, L. Litov, B. Pavlov, P. Petkov, W. Fang, M. Ahmad, J. G. Bian, G. M. Chen, H. S. Chen, M. Chen, Y. Chen, T. Cheng, C. H. Jiang, D. Leggat, Z. Liu, F. Romeo, S. M. Shaheen, A. Spiezia, J. Tao, C. Wang, Z. Wang, H. Zhang, J. Zhao, Y. Ban, G. Chen, Q. Li, S. Liu, Y. Mao, S. J. Qian, D. Wang, Z. Xu, C. Avila, A. Cabrera, L. F. Chaparro Sierra, C. Florez, J. P. Gomez, C. F. González Hernández, J. D. Ruiz Alvarez, J. C. Sanabria, N. Godinovic, D. Lelas, I. Puljak, P. M. Ribeiro Cipriano, T. Sculac, Z. Antunovic, M. Kovac, V. Brigljevic, D. Ferencek, K. Kadija, S. Micanovic, L. Sudic, T. Susa, A. Attikis, G. Mavromanolakis, J. Mousa, C. Nicolaou, F. Ptochos, P. A. Razis, H. Rykaczewski, D. Tsiakkouri, M. Finger, M. Finger, E. Carrera Jarrin, Y. Assran, T. Elkafrawy, A. Mahrous, B. Calpas, M. Kadastik, M. Murumaa, L. Perrini, M. Raidal, A. Tiko, C. Veelken, P. Eerola, J. Pekkanen, M. Voutilainen, J. Härkönen, T. Järvinen, V. Karimäki, R. Kinnunen, T. Lampén, K. Lassila-Perini, S. Lehti, T. Lindén, P. Luukka, J. Tuominiemi, E. Tuovinen, L. Wendland, J. Talvitie, T. Tuuva, M. Besancon, F. Couderc, M. Dejardin, D. Denegri, B. Fabbro, J. L. Faure, C. Favaro, F. Ferri, S. Ganjour, S. Ghosh, A. Givernaud, P. Gras, G. Hamel de Monchenault, P. Jarry, I. Kucher, E. Locci, M. Machet, J. Malcles, J. Rander, A. Rosowsky, M. Titov, A. Zghiche, A. Abdulsalam, I. Antropov, S. Baffioni, F. Beaudette, P. Busson, L. Cadamuro, E. Chapon, C. Charlot, O. Davignon, R. Granier de Cassagnac, M. Jo, S. Lisniak, P. Miné, M. Nguyen, C. Ochando, G. Ortona, P. Paganini, P. Pigard, S. Regnard, R. Salerno, Y. Sirois, T. Strebler, Y. Yilmaz, A. Zabi, J.-L. Agram, J. Andrea, A. Aubin, D. Bloch, J.-M. Brom, M. Buttignol, E. C. Chabert, N. Chanon, C. Collard, E. Conte, X. Coubez, J.-C. Fontaine, D. Gelé, U. Goerlach, A.-C. Le Bihan, K. Skovpen, P. Van Hove, S. Gadrat, S. Beauceron, C. Bernet, G. Boudoul, E. Bouvier, C. A. Carrillo Montoya, R. Chierici, D. Contardo, B. Courbon, P. Depasse, H. El Mamouni, J. Fan, J. Fay, S. Gascon, M. Gouzevitch, G. Grenier, B. Ille, F. Lagarde, I. B. Laktineh, M. Lethuillier, L. Mirabito, A. L. Pequegnot, S. Perries, A. Popov, D. Sabes, V. Sordini, M. Vander Donckt, P. Verdier, S. Viret, T. Toriashvili, Z. Tsamalaidze, C. Autermann, S. Beranek, L. Feld, A. Heister, M. K. Kiesel, K. Klein, M. Lipinski, A. Ostapchuk, M. Preuten, F. Raupach, S. Schael, C. Schomakers, J. Schulz, T. Verlage, H. Weber, V. Zhukov, A. Albert, M. Brodski, E. Dietz-Laursonn, D. Duchardt, M. Endres, M. Erdmann, S. Erdweg, T. Esch, R. Fischer, A. Güth, M. Hamer, T. Hebbeker, C. Heidemann, K. Hoepfner, S. Knutzen, M. Merschmeyer, A. Meyer, P. Millet, S. Mukherjee, M. Olschewski, K. Padeken, T. Pook, M. Radziej, H. Reithler, M. Rieger, F. Scheuch, L. Sonnenschein, D. Teyssier, S. Thüer, V. Cherepanov, G. Flügge, F. Hoehle, B. Kargoll, T. Kress, A. Künsken, J. Lingemann, T. Müller, A. Nehrkorn, A. Nowack, I. M. Nugent, C. Pistone, O. Pooth, A. Stahl, M. Aldaya Martin, T. Arndt, C. Asawatangtrakuldee, K. Beernaert, O. Behnke, U. Behrens, A. A. Bin Anuar, K. Borras, A. Campbell, P. Connor, C. Contreras-Campana, F. Costanza, C. Diez Pardos, G. Dolinska, G. Eckerlin, D. Eckstein, T. Eichhorn, E. Eren, E. Gallo, J. Garay Garcia, A. Geiser, A. Gizhko, J. M. Grados Luyando, P. Gunnellini, A. Harb, J. Hauk, M. Hempel, H. Jung, A. Kalogeropoulos, O. Karacheban, M. Kasemann, J. Keaveney, C. Kleinwort, I. Korol, D. Krücker, W. Lange, A. Lelek, J. Leonard, K. Lipka, A. Lobanov, W. Lohmann, R. Mankel, I.-A. Melzer-Pellmann, A. B. Meyer, G. Mittag, J. Mnich, A. Mussgiller, E. Ntomari, D. Pitzl, R. Placakyte, A. Raspereza, B. Roland, M. Ö. Sahin, P. Saxena, T. Schoerner-Sadenius, C. Seitz, S. Spannagel, N. Stefaniuk, G. P. Van Onsem, R. Walsh, C. Wissing, V. Blobel, M. Centis Vignali, A. R. Draeger, T. Dreyer, E. Garutti, D. Gonzalez, J. Haller, M. Hoffmann, A. Junkes, R. Klanner, R. Kogler, N. Kovalchuk, T. Lapsien, T. Lenz, I. Marchesini, D. Marconi, M. Meyer, M. Niedziela, D. Nowatschin, F. Pantaleo, T. Peiffer, A. Perieanu, J. Poehlsen, C. Sander, C. Scharf, P. Schleper, A. Schmidt, S. Schumann, J. Schwandt, H. Stadie, G. Steinbrück, F. M. Stober, M. Stöver, H. Tholen, D. Troendle, E. Usai, L. Vanelderen, A. Vanhoefer, B. Vormwald, M. Akbiyik, C. Barth, S. Baur, C. Baus, J. Berger, E. Butz, R. Caspart, T. Chwalek, F. Colombo, W. De Boer, A. Dierlamm, S. Fink, B. Freund, R. Friese, M. Giffels, A. Gilbert, P. Goldenzweig, D. Haitz, F. Hartmann, S. M. Heindl, U. Husemann, I. Katkov, S. Kudella, P. Lobelle Pardo, H. Mildner, M. U. Mozer, Th. Müller, M. Plagge, G. Quast, K. Rabbertz, S. Röcker, F. Roscher, M. Schröder, I. Shvetsov, G. Sieber, H. J. Simonis, R. Ulrich, J. Wagner-Kuhr, S. Wayand, M. Weber, T. Weiler, S. Williamson, C. Wöhrmann, R. Wolf, G. Anagnostou, G. Daskalakis, T. Geralis, V. A. Giakoumopoulou, A. Kyriakis, D. Loukas, I. Topsis-Giotis, S. Kesisoglou, A. Panagiotou, N. Saoulidou, E. Tziaferi, I. Evangelou, G. Flouris, C. Foudas, P. Kokkas, N. Loukas, N. Manthos, I. Papadopoulos, E. Paradas, N. Filipovic, G. Bencze, C. Hajdu, P. Hidas, D. Horvath, F. Sikler, V. Veszpremi, G. Vesztergombi, A. J. Zsigmond, N. Beni, S. Czellar, J. Karancsi, A. Makovec, J. Molnar, Z. Szillasi, M. Bartók, P. Raics, Z. L. Trocsanyi, B. Ujvari, S. Bahinipati, S. Choudhury, P. Mal, K. Mandal, A. Nayak, D. K. Sahoo, N. Sahoo, S. K. Swain, S. Bansal, S. B. Beri, V. Bhatnagar, R. Chawla, U. Bhawandeep, A. K. Kalsi, A. Kaur, M. Kaur, R. Kumar, P. Kumari, A. Mehta, M. Mittal, J. B. Singh, G. Walia, Ashok Kumar, A. Bhardwaj, B. C. Choudhary, R. B. Garg, S. Keshri, S. Malhotra, M. Naimuddin, N. Nishu, K. Ranjan, R. Sharma, V. Sharma, R. Bhattacharya, S. Bhattacharya, K. Chatterjee, S. Dey, S. Dutt, S. Dutta, S. Ghosh, N. Majumdar, A. Modak, K. Mondal, S. Mukhopadhyay, S. Nandan, A. Purohit, A. Roy, D. Roy, S. Roy Chowdhury, S. Sarkar, M. Sharan, S. Thakur, P. K. Behera, R. Chudasama, D. Dutta, V. Jha, V. Kumar, A. K. Mohanty, P. K. Netrakanti, L. M. Pant, P. Shukla, A. Topkar, T. Aziz, S. Dugad, G. Kole, B. Mahakud, S. Mitra, G. B. Mohanty, B. Parida, N. Sur, B. Sutar, S. Banerjee, S. Bhowmik, R. K. Dewanjee, S. Ganguly, M. Guchait, Sa. Jain, S. Kumar, M. Maity, G. Majumder, K. Mazumdar, T. Sarkar, N. Wickramage, S. Chauhan, S. Dube, V. Hegde, A. Kapoor, K. Kothekar, A. Rane, S. Sharma, H. Behnamian, S. Chenarani, E. Eskandari Tadavani, S. M. Etesami, A. Fahim, M. Khakzad, M. Mohammadi Najafabadi, M. Naseri, S. Paktinat Mehdiabadi, F. Rezaei Hosseinabadi, B. Safarzadeh, M. Zeinali, M. Felcini, M. Grunewald, M. Abbrescia, C. Calabria, C. Caputo, A. Colaleo, D. Creanza, L. Cristella, N. De Filippis, M. De Palma, L. Fiore, G. Iaselli, G. Maggi, M. Maggi, G. Miniello, S. My, S. Nuzzo, A. Pompili, G. Pugliese, R. Radogna, A. Ranieri, G. Selvaggi, L. Silvestris, R. Venditti, P. Verwilligen, G. Abbiendi, C. Battilana, D. Bonacorsi, S. Braibant-Giacomelli, L. Brigliadori, R. Campanini, P. Capiluppi, A. Castro, F. R. Cavallo, S. S. Chhibra, G. Codispoti, M. Cuffiani, G. M. Dallavalle, F. Fabbri, A. Fanfani, D. Fasanella, P. Giacomelli, C. Grandi, L. Guiducci, S. Marcellini, G. Masetti, A. Montanari, F. L. Navarria, A. Perrotta, A. M. Rossi, T. Rovelli, G. P. Siroli, N. Tosi, S. Albergo, M. Chiorboli, S. Costa, A. Di Mattia, F. Giordano, R. Potenza, A. Tricomi, C. Tuve, G. Barbagli, V. Ciulli, C. Civinini, R. D’Alessandro, E. Focardi, V. Gori, P. Lenzi, M. Meschini, S. Paoletti, G. Sguazzoni, L. Viliani, L. Benussi, S. Bianco, F. Fabbri, D. Piccolo, F. Primavera, V. Calvelli, F. Ferro, M. Lo Vetere, M. R. Monge, E. Robutti, S. Tosi, L. Brianza, M. E. Dinardo, S. Fiorendi, S. Gennai, A. Ghezzi, P. Govoni, M. Malberti, S. Malvezzi, R. A. Manzoni, D. Menasce, L. Moroni, M. Paganoni, D. Pedrini, S. Pigazzini, S. Ragazzi, T. Tabarelli de Fatis, S. Buontempo, N. Cavallo, G. De Nardo, S. Di Guida, M. Esposito, F. Fabozzi, F. Fienga, A. O. M. Iorio, G. Lanza, L. Lista, S. Meola, P. Paolucci, C. Sciacca, F. Thyssen, P. Azzi, N. Bacchetta, L. Benato, D. Bisello, A. Boletti, R. Carlin, A. Carvalho Antunes De Oliveira, P. Checchia, M. Dall’Osso, P. De Castro Manzano, T. Dorigo, U. Dosselli, F. Gasparini, U. Gasparini, A. Gozzelino, S. Lacaprara, M. Margoni, A. T. Meneguzzo, J. Pazzini, N. Pozzobon, P. Ronchese, F. Simonetto, E. Torassa, M. Zanetti, P. Zotto, G. Zumerle, A. Braghieri, A. Magnani, P. Montagna, S. P. Ratti, V. Re, C. Riccardi, P. Salvini, I. Vai, P. Vitulo, L. Alunni Solestizi, G. M. Bilei, D. Ciangottini, L. Fanò, P. Lariccia, R. Leonardi, G. Mantovani, M. Menichelli, A. Saha, A. Santocchia, K. Androsov, P. Azzurri, G. Bagliesi, J. Bernardini, T. Boccali, R. Castaldi, M. A. Ciocci, R. Dell’Orso, S. Donato, G. Fedi, A. Giassi, M. T. Grippo, F. Ligabue, T. Lomtadze, L. Martini, A. Messineo, F. Palla, A. Rizzi, A. Savoy-Navarro, P. Spagnolo, R. Tenchini, G. Tonelli, A. Venturi, P. G. Verdini, L. Barone, F. Cavallari, M. Cipriani, D. Del Re, M. Diemoz, S. Gelli, E. Longo, F. Margaroli, B. Marzocchi, P. Meridiani, G. Organtini, R. Paramatti, F. Preiato, S. Rahatlou, C. Rovelli, F. Santanastasio, N. Amapane, R. Arcidiacono, S. Argiro, M. Arneodo, N. Bartosik, R. Bellan, C. Biino, N. Cartiglia, F. Cenna, M. Costa, R. Covarelli, A. Degano, N. Demaria, L. Finco, B. Kiani, C. Mariotti, S. Maselli, E. Migliore, V. Monaco, E. Monteil, M. M. Obertino, L. Pacher, N. Pastrone, M. Pelliccioni, G. L. Pinna Angioni, F. Ravera, A. Romero, M. Ruspa, R. Sacchi, K. Shchelina, V. Sola, A. Solano, A. Staiano, P. Traczyk, S. Belforte, M. Casarsa, F. Cossutti, G. Della Ricca, A. Zanetti, D. H. Kim, G. N. Kim, M. S. Kim, S. Lee, S. W. Lee, Y. D. Oh, S. Sekmen, D. C. Son, Y. C. Yang, A. Lee, H. Kim, J. A. Brochero Cifuentes, T. J. Kim, S. Cho, S. Choi, Y. Go, D. Gyun, S. Ha, B. Hong, Y. Jo, Y. Kim, B. Lee, K. Lee, K. S. Lee, S. Lee, J. Lim, S. K. Park, Y. Roh, J. Almond, J. Kim, H. Lee, S. B. Oh, B. C. Radburn-Smith, S. h. Seo, U. K. Yang, H. D. Yoo, G. B. Yu, M. Choi, H. Kim, J. H. Kim, J. S. H. Lee, I. C. Park, G. Ryu, M. S. Ryu, Y. Choi, J. Goh, C. Hwang, J. Lee, I. Yu, V. Dudenas, A. Juodagalvis, J. Vaitkus, I. Ahmed, Z. A. Ibrahim, J. R. Komaragiri, M. A. B. Md Ali, F. Mohamad Idris, W. A. T. Wan Abdullah, M. N. Yusli, Z. Zolkapli, H. Castilla-Valdez, E. De La Cruz-Burelo, I. Heredia-De La Cruz, A. Hernandez-Almada, R. Lopez-Fernandez, R. Magaña Villalba, J. Mejia Guisao, A. Sanchez-Hernandez, S. Carrillo Moreno, C. Oropeza Barrera, F. Vazquez Valencia, S. Carpinteyro, I. Pedraza, H. A. Salazar Ibarguen, C. Uribe Estrada, A. Morelos Pineda, D. Krofcheck, P. H. Butler, A. Ahmad, M. Ahmad, Q. Hassan, H. R. Hoorani, W. A. Khan, A. Saddique, M. A. Shah, M. Shoaib, M. Waqas, H. Bialkowska, M. Bluj, B. Boimska, T. Frueboes, M. Górski, M. Kazana, K. Nawrocki, K. Romanowska-Rybinska, M. Szleper, P. Zalewski, K. Bunkowski, A. Byszuk, K. Doroba, A. Kalinowski, M. Konecki, J. Krolikowski, M. Misiura, M. Olszewski, M. Walczak, P. Bargassa, C. Beirão Da Cruz E Silva, A. Di Francesco, P. Faccioli, P. G. Ferreira Parracho, M. Gallinaro, J. Hollar, N. Leonardo, L. Lloret Iglesias, M. V. Nemallapudi, J. Rodrigues Antunes, J. Seixas, O. Toldaiev, D. Vadruccio, J. Varela, P. Vischia, V. Alexakhin, M. Gavrilenko, I. Golutvin, A. Kamenev, V. Karjavin, V. Korenkov, A. Lanev, A. Malakhov, V. Matveev, V. V. Mitsyn, V. Palichik, V. Perelygin, S. Shmatov, S. Shulha, N. Skatchkov, V. Smirnov, E. Tikhonenko, A. Zarubin, L. Chtchipounov, V. Golovtsov, Y. Ivanov, V. Kim, E. Kuznetsova, V. Murzin, V. Oreshkin, V. Sulimov, A. Vorobyev, Yu. Andreev, A. Dermenev, S. Gninenko, N. Golubev, A. Karneyeu, M. Kirsanov, N. Krasnikov, A. Pashenkov, D. Tlisov, A. Toropin, V. Epshteyn, V. Gavrilov, N. Lychkovskaya, V. Popov, I. Pozdnyakov, G. Safronov, A. Spiridonov, M. Toms, E. Vlasov, A. Zhokin, A. Bylinkin, M. Chadeeva, O. Markin, E. Popova, V. Andreev, M. Azarkin, I. Dremin, M. Kirakosyan, A. Leonidov, S. V. Rusakov, A. Terkulov, A. Baskakov, A. Belyaev, E. Boos, V. Bunichev, M. Dubinin, L. Dudko, A. Ershov, V. Klyukhin, N. Korneeva, I. Lokhtin, I. Miagkov, S. Obraztsov, M. Perfilov, S. Petrushanko, V. Savrin, V. Blinov, Y. Skovpen, I. Azhgirey, I. Bayshev, S. Bitioukov, D. Elumakhov, V. Kachanov, A. Kalinin, D. Konstantinov, V. Krychkine, V. Petrov, R. Ryutin, A. Sobol, S. Troshin, N. Tyurin, A. Uzunian, A. Volkov, P. Adzic, P. Cirkovic, D. Devetak, M. Dordevic, J. Milosevic, V. Rekovic, J. Alcaraz Maestre, M. Barrio Luna, E. Calvo, M. Cerrada, M. Chamizo Llatas, N. Colino, B. De La Cruz, A. Delgado Peris, A. Escalante Del Valle, C. Fernandez Bedoya, J. P. Fernández Ramos, J. Flix, M. C. Fouz, P. Garcia-Abia, O. Gonzalez Lopez, S. Goy Lopez, J. M. Hernandez, M. I. Josa, E. Navarro De Martino, A. Pérez-Calero Yzquierdo, J. Puerta Pelayo, A. Quintario Olmeda, I. Redondo, L. Romero, M. S. Soares, J. F. de Trocóniz, M. Missiroli, D. Moran, J. Cuevas, J. Fernandez Menendez, I. Gonzalez Caballero, J. R. González Fernández, E. Palencia Cortezon, S. Sanchez Cruz, I. Suárez Andrés, J. M. Vizan Garcia, I. J. Cabrillo, A. Calderon, J. R. Castiñeiras De Saa, E. Curras, M. Fernandez, J. Garcia-Ferrero, G. Gomez, A. Lopez Virto, J. Marco, C. Martinez Rivero, F. Matorras, J. Piedra Gomez, T. Rodrigo, A. Ruiz-Jimeno, L. Scodellaro, N. Trevisani, I. Vila, R. Vilar Cortabitarte, D. Abbaneo, E. Auffray, G. Auzinger, M. Bachtis, P. Baillon, A. H. Ball, D. Barney, P. Bloch, A. Bocci, A. Bonato, C. Botta, T. Camporesi, R. Castello, M. Cepeda, G. Cerminara, M. D’Alfonso, D. d’Enterria, A. Dabrowski, V. Daponte, A. David, M. De Gruttola, A. De Roeck, E. Di Marco, M. Dobson, B. Dorney, T. du Pree, D. Duggan, M. Dünser, N. Dupont, A. Elliott-Peisert, S. Fartoukh, G. Franzoni, J. Fulcher, W. Funk, D. Gigi, K. Gill, M. Girone, F. Glege, D. Gulhan, S. Gundacker, M. Guthoff, J. Hammer, P. Harris, J. Hegeman, V. Innocente, P. Janot, J. Kieseler, H. Kirschenmann, V. Knünz, A. Kornmayer, M. J. Kortelainen, K. Kousouris, M. Krammer, C. Lange, P. Lecoq, C. Lourenço, M. T. Lucchini, L. Malgeri, M. Mannelli, A. Martelli, F. Meijers, J. A. Merlin, S. Mersi, E. Meschi, P. Milenovic, F. Moortgat, S. Morovic, M. Mulders, H. Neugebauer, S. Orfanelli, L. Orsini, L. Pape, E. Perez, M. Peruzzi, A. Petrilli, G. Petrucciani, A. Pfeiffer, M. Pierini, A. Racz, T. Reis, G. Rolandi, M. Rovere, M. Ruan, H. Sakulin, J. B. Sauvan, C. Schäfer, C. Schwick, M. Seidel, A. Sharma, P. Silva, P. Sphicas, J. Steggemann, M. Stoye, Y. Takahashi, M. Tosi, D. Treille, A. Triossi, A. Tsirou, V. Veckalns, G. I. Veres, N. Wardle, H. K. Wöhri, A. Zagozdzinska, W. D. Zeuner, W. Bertl, K. Deiters, W. Erdmann, R. Horisberger, Q. Ingram, H. C. Kaestli, D. Kotlinski, U. Langenegger, T. Rohe, F. Bachmair, L. Bäni, L. Bianchini, B. Casal, G. Dissertori, M. Dittmar, M. Donegà, C. Grab, C. Heidegger, D. Hits, J. Hoss, G. Kasieczka, P. Lecomte, W. Lustermann, B. Mangano, M. Marionneau, P. Martinez Ruiz del Arbol, M. Masciovecchio, M. T. Meinhard, D. Meister, F. Micheli, P. Musella, F. Nessi-Tedaldi, F. Pandolfi, J. Pata, F. Pauss, G. Perrin, L. Perrozzi, M. Quittnat, M. Rossini, M. Schönenberger, A. Starodumov, V. R. Tavolaro, K. Theofilatos, R. Wallny, T. K. Aarrestad, C. Amsler, L. Caminada, M. F. Canelli, A. De Cosa, C. Galloni, A. Hinzmann, T. Hreus, B. Kilminster, J. Ngadiuba, D. Pinna, G. Rauco, P. Robmann, D. Salerno, Y. Yang, A. Zucchetta, V. Candelise, T. H. Doan, Sh. Jain, R. Khurana, M. Konyushikhin, C. M. Kuo, W. Lin, Y. J. Lu, A. Pozdnyakov, S. S. Yu, Arun Kumar, P. Chang, Y. H. Chang, Y. W. Chang, Y. Chao, K. F. Chen, P. H. Chen, C. Dietz, F. Fiori, W.-S. Hou, Y. Hsiung, Y. F. Liu, R.-S. Lu, M. Miñano Moya, E. Paganis, A. Psallidas, J. F. Tsai, Y. M. Tzeng, B. Asavapibhop, G. Singh, N. Srimanobhas, N. Suwonjandee, A. Adiguzel, M. N. Bakirci, S. Cerci, S. Damarseckin, Z. S. Demiroglu, C. Dozen, I. Dumanoglu, S. Girgis, G. Gokbulut, Y. Guler, I. Hos, E. E. Kangal, O. Kara, A. Kayis Topaksu, U. Kiminsu, M. Oglakci, G. Onengut, K. Ozdemir, B. Tali, S. Turkcapar, I. S. Zorbakir, C. Zorbilmez, B. Bilin, S. Bilmis, B. Isildak, G. Karapinar, M. Yalvac, M. Zeyrek, E. Gülmez, M. Kaya, O. Kaya, E. A. Yetkin, T. Yetkin, A. Cakir, K. Cankocak, S. Sen, B. Grynyov, L. Levchuk, P. Sorokin, R. Aggleton, F. Ball, L. Beck, J. J. Brooke, D. Burns, E. Clement, D. Cussans, H. Flacher, J. Goldstein, M. Grimes, G. P. Heath, H. F. Heath, J. Jacob, L. Kreczko, C. Lucas, D. M. Newbold, S. Paramesvaran, A. Poll, T. Sakuma, S. Seif El Nasr-storey, D. Smith, V. J. Smith, K. W. Bell, A. Belyaev, C. Brew, R. M. Brown, L. Calligaris, D. Cieri, D. J. A. Cockerill, J. A. Coughlan, K. Harder, S. Harper, E. Olaiya, D. Petyt, C. H. Shepherd-Themistocleous, A. Thea, I. R. Tomalin, T. Williams, M. Baber, R. Bainbridge, O. Buchmuller, A. Bundock, D. Burton, S. Casasso, M. Citron, D. Colling, L. Corpe, P. Dauncey, G. Davies, A. De Wit, M. Della Negra, R. Di Maria, P. Dunne, A. Elwood, D. Futyan, Y. Haddad, G. Hall, G. Iles, T. James, R. Lane, C. Laner, R. Lucas, L. Lyons, A.-M. Magnan, S. Malik, L. Mastrolorenzo, J. Nash, A. Nikitenko, J. Pela, B. Penning, M. Pesaresi, D. M. Raymond, A. Richards, A. Rose, C. Seez, S. Summers, A. Tapper, K. Uchida, M. Vazquez Acosta, T. Virdee, J. Wright, S. C. Zenz, J. E. Cole, P. R. Hobson, A. Khan, P. Kyberd, D. Leslie, I. D. Reid, P. Symonds, L. Teodorescu, M. Turner, A. Borzou, K. Call, J. Dittmann, K. Hatakeyama, H. Liu, N. Pastika, O. Charaf, S. I. Cooper, C. Henderson, P. Rumerio, C. West, D. Arcaro, A. Avetisyan, T. Bose, D. Gastler, D. Rankin, C. Richardson, J. Rohlf, L. Sulak, D. Zou, G. Benelli, E. Berry, D. Cutts, A. Garabedian, J. Hakala, U. Heintz, J. M. Hogan, O. Jesus, K. H. M. Kwok, E. Laird, G. Landsberg, Z. Mao, M. Narain, S. Piperov, S. Sagir, E. Spencer, R. Syarif, R. Breedon, G. Breto, D. Burns, M. Calderon De La Barca Sanchez, S. Chauhan, M. Chertok, J. Conway, R. Conway, P. T. Cox, R. Erbacher, C. Flores, G. Funk, M. Gardner, W. Ko, R. Lander, C. Mclean, M. Mulhearn, D. Pellett, J. Pilot, S. Shalhout, J. Smith, M. Squires, D. Stolp, M. Tripathi, S. Wilbur, R. Yohay, C. Bravo, R. Cousins, P. Everaerts, A. Florent, J. Hauser, M. Ignatenko, N. Mccoll, D. Saltzberg, C. Schnaible, E. Takasugi, V. Valuev, M. Weber, K. Burt, R. Clare, J. Ellison, J. W. Gary, S. M. A. Ghiasi Shirazi, G. Hanson, J. Heilman, P. Jandir, E. Kennedy, F. Lacroix, O. R. Long, M. Olmedo Negrete, M. I. Paneva, A. Shrinivas, W. Si, H. Wei, S. Wimpenny, B. R. Yates, J. G. Branson, G. B. Cerati, S. Cittolin, M. Derdzinski, R. Gerosa, A. Holzner, D. Klein, V. Krutelyov, J. Letts, I. Macneill, D. Olivito, S. Padhi, M. Pieri, M. Sani, V. Sharma, S. Simon, M. Tadel, A. Vartak, S. Wasserbaech, C. Welke, J. Wood, F. Würthwein, A. Yagil, G. Zevi Della Porta, N. Amin, R. Bhandari, J. Bradmiller-Feld, C. Campagnari, A. Dishaw, V. Dutta, K. Flowers, M. Franco Sevilla, P. Geffert, C. George, F. Golf, L. Gouskos, J. Gran, R. Heller, J. Incandela, S. D. Mullin, A. Ovcharova, J. Richman, D. Stuart, I. Suarez, J. Yoo, D. Anderson, A. Apresyan, J. Bendavid, A. Bornheim, J. Bunn, Y. Chen, J. Duarte, J. M. Lawhorn, A. Mott, H. B. Newman, C. Pena, M. Spiropulu, J. R. Vlimant, S. Xie, R. Y. Zhu, M. B. Andrews, V. Azzolini, T. Ferguson, M. Paulini, J. Russ, M. Sun, H. Vogel, I. Vorobiev, M. Weinberg, J. P. Cumalat, W. T. Ford, F. Jensen, A. Johnson, M. Krohn, T. Mulholland, K. Stenson, S. R. Wagner, J. Alexander, J. Chaves, J. Chu, S. Dittmer, K. Mcdermott, N. Mirman, G. Nicolas Kaufman, J. R. Patterson, A. Rinkevicius, A. Ryd, L. Skinnari, L. Soffi, S. M. Tan, Z. Tao, J. Thom, J. Tucker, P. Wittich, M. Zientek, D. Winn, S. Abdullin, M. Albrow, G. Apollinari, S. Banerjee, L. A. T. Bauerdick, A. Beretvas, J. Berryhill, P. C. Bhat, G. Bolla, K. Burkett, J. N. Butler, H. W. K. Cheung, F. Chlebana, S. Cihangir, M. Cremonesi, V. D. Elvira, I. Fisk, J. Freeman, E. Gottschalk, L. Gray, D. Green, S. Grünendahl, O. Gutsche, D. Hare, R. M. Harris, S. Hasegawa, J. Hirschauer, Z. Hu, B. Jayatilaka, S. Jindariani, M. Johnson, U. Joshi, B. Klima, B. Kreis, S. Lammel, J. Linacre, D. Lincoln, R. Lipton, M. Liu, T. Liu, R. Lopes De Sá, J. Lykken, K. Maeshima, N. Magini, J. M. Marraffino, S. Maruyama, D. Mason, P. McBride, P. Merkel, S. Mrenna, S. Nahn, C. Newman-Holmes, V. O’Dell, K. Pedro, O. Prokofyev, G. Rakness, L. Ristori, E. Sexton-Kennedy, A. Soha, W. J. Spalding, L. Spiegel, S. Stoynev, J. Strait, N. Strobbe, L. Taylor, S. Tkaczyk, N. V. Tran, L. Uplegger, E. W. Vaandering, C. Vernieri, M. Verzocchi, R. Vidal, M. Wang, H. A. Weber, A. Whitbeck, D. Acosta, P. Avery, P. Bortignon, D. Bourilkov, A. Brinkerhoff, A. Carnes, M. Carver, D. Curry, S. Das, R. D. Field, I. K. Furic, J. Konigsberg, A. Korytov, J. F. Low, P. Ma, K. Matchev, H. Mei, G. Mitselmakher, D. Rank, L. Shchutska, D. Sperka, L. Thomas, J. Wang, S. Wang, J. Yelton, S. Linn, P. Markowitz, G. Martinez, J. L. Rodriguez, A. Ackert, J. R. Adams, T. Adams, A. Askew, S. Bein, B. Diamond, S. Hagopian, V. Hagopian, K. F. Johnson, A. Khatiwada, H. Prosper, A. Santra, M. M. Baarmand, V. Bhopatkar, S. Colafranceschi, M. Hohlmann, D. Noonan, T. Roy, F. Yumiceva, M. R. Adams, L. Apanasevich, D. Berry, R. R. Betts, I. Bucinskaite, R. Cavanaugh, O. Evdokimov, L. Gauthier, C. E. Gerber, D. J. Hofman, K. Jung, P. Kurt, C. O’Brien, I. D. Sandoval Gonzalez, P. Turner, N. Varelas, H. Wang, Z. Wu, M. Zakaria, J. Zhang, B. Bilki, W. Clarida, K. Dilsiz, S. Durgut, R. P. Gandrajula, M. Haytmyradov, V. Khristenko, J.-P. Merlo, H. Mermerkaya, A. Mestvirishvili, A. Moeller, J. Nachtman, H. Ogul, Y. Onel, F. Ozok, A. Penzo, C. Snyder, E. Tiras, J. Wetzel, K. Yi, I. Anderson, B. Blumenfeld, A. Cocoros, N. Eminizer, D. Fehling, L. Feng, A. V. Gritsan, P. Maksimovic, C. Martin, M. Osherson, J. Roskes, U. Sarica, M. Swartz, M. Xiao, Y. Xin, C. You, A. Al-Bataineh, P. Baringer, A. Bean, S. Boren, J. Bowen, C. Bruner, J. Castle, L. Forthomme, R. P. Kenny, A. Kropivnitskaya, D. Majumder, W. Mcbrayer, M. Murray, S. Sanders, R. Stringer, J. D. Tapia Takaki, Q. Wang, A. Ivanov, K. Kaadze, S. Khalil, Y. Maravin, A. Mohammadi, L. K. Saini, N. Skhirtladze, S. Toda, F. Rebassoo, D. Wright, C. Anelli, A. Baden, O. Baron, A. Belloni, B. Calvert, S. C. Eno, C. Ferraioli, J. A. Gomez, N. J. Hadley, S. Jabeen, R. G. Kellogg, T. Kolberg, J. Kunkle, Y. Lu, A. C. Mignerey, F. Ricci-Tam, Y. H. Shin, A. Skuja, M. B. Tonjes, S. C. Tonwar, D. Abercrombie, B. Allen, A. Apyan, R. Barbieri, A. Baty, R. Bi, K. Bierwagen, S. Brandt, W. Busza, I. A. Cali, Z. Demiragli, L. Di Matteo, G. Gomez Ceballos, M. Goncharov, D. Hsu, Y. Iiyama, G. M. Innocenti, M. Klute, D. Kovalskyi, K. Krajczar, Y. S. Lai, Y.-J. Lee, A. Levin, P. D. Luckey, B. Maier, A. C. Marini, C. Mcginn, C. Mironov, S. Narayanan, X. Niu, C. Paus, C. Roland, G. Roland, J. Salfeld-Nebgen, G. S. F. Stephans, K. Sumorok, K. Tatar, M. Varma, D. Velicanu, J. Veverka, J. Wang, T. W. Wang, B. Wyslouch, M. Yang, V. Zhukova, A. C. Benvenuti, R. M. Chatterjee, A. Evans, A. Finkel, A. Gude, P. Hansen, S. Kalafut, S. C. Kao, Y. Kubota, Z. Lesko, J. Mans, S. Nourbakhsh, N. Ruckstuhl, R. Rusack, N. Tambe, J. Turkewitz, J. G. Acosta, S. Oliveros, E. Avdeeva, R. Bartek, K. Bloom, D. R. Claes, A. Dominguez, C. Fangmeier, R. Gonzalez Suarez, R. Kamalieddin, I. Kravchenko, A. Malta Rodrigues, F. Meier, J. Monroy, J. E. Siado, G. R. Snow, B. Stieger, M. Alyari, J. Dolen, J. George, A. Godshalk, C. Harrington, I. Iashvili, J. Kaisen, A. Kharchilava, A. Kumar, A. Parker, S. Rappoccio, B. Roozbahani, G. Alverson, E. Barberis, A. Hortiangtham, A. Massironi, D. M. Morse, D. Nash, T. Orimoto, R. Teixeira De Lima, D. Trocino, R.-J. Wang, D. Wood, S. Bhattacharya, K. A. Hahn, A. Kubik, A. Kumar, N. Mucia, N. Odell, B. Pollack, M. H. Schmitt, K. Sung, M. Trovato, M. Velasco, N. Dev, M. Hildreth, K. Hurtado Anampa, C. Jessop, D. J. Karmgard, N. Kellams, K. Lannon, N. Marinelli, F. Meng, C. Mueller, Y. Musienko, M. Planer, A. Reinsvold, R. Ruchti, G. Smith, S. Taroni, M. Wayne, M. Wolf, A. Woodard, J. Alimena, L. Antonelli, J. Brinson, B. Bylsma, L. S. Durkin, S. Flowers, B. Francis, A. Hart, C. Hill, R. Hughes, W. Ji, B. Liu, W. Luo, D. Puigh, B. L. Winer, H. W. Wulsin, S. Cooperstein, O. Driga, P. Elmer, J. Hardenbrook, P. Hebda, D. Lange, J. Luo, D. Marlow, J. Mc Donald, T. Medvedeva, K. Mei, M. Mooney, J. Olsen, C. Palmer, P. Piroué, D. Stickland, C. Tully, A. Zuranski, S. Malik, A. Barker, V. E. Barnes, S. Folgueras, L. Gutay, M. K. Jha, M. Jones, A. W. Jung, D. H. Miller, N. Neumeister, J. F. Schulte, X. Shi, J. Sun, A. Svyatkovskiy, F. Wang, W. Xie, L. Xu, N. Parashar, J. Stupak, A. Adair, B. Akgun, Z. Chen, K. M. Ecklund, F. J. M. Geurts, M. Guilbaud, W. Li, B. Michlin, M. Northup, B. P. Padley, R. Redjimi, J. Roberts, J. Rorie, Z. Tu, J. Zabel, B. Betchart, A. Bodek, P. de Barbaro, R. Demina, Y. t. Duh, T. Ferbel, M. Galanti, A. Garcia-Bellido, J. Han, O. Hindrichs, A. Khukhunaishvili, K. H. Lo, P. Tan, M. Verzetti, A. Agapitos, J. P. Chou, E. Contreras-Campana, Y. Gershtein, T. A. Gómez Espinosa, E. Halkiadakis, M. Heindl, D. Hidas, E. Hughes, S. Kaplan, R. Kunnawalkam Elayavalli, S. Kyriacou, A. Lath, K. Nash, H. Saka, S. Salur, S. Schnetzer, D. Sheffield, S. Somalwar, R. Stone, S. Thomas, P. Thomassen, M. Walker, A. G. Delannoy, M. Foerster, J. Heideman, G. Riley, K. Rose, S. Spanier, K. Thapa, O. Bouhali, A. Celik, M. Dalchenko, M. De Mattia, A. Delgado, S. Dildick, R. Eusebi, J. Gilmore, T. Huang, E. Juska, T. Kamon, R. Mueller, Y. Pakhotin, R. Patel, A. Perloff, L. Perniè, D. Rathjens, A. Rose, A. Safonov, A. Tatarinov, K. A. Ulmer, N. Akchurin, C. Cowden, J. Damgov, F. De Guio, C. Dragoiu, P. R. Dudero, J. Faulkner, E. Gurpinar, S. Kunori, K. Lamichhane, S. W. Lee, T. Libeiro, T. Peltola, S. Undleeb, I. Volobouev, Z. Wang, S. Greene, A. Gurrola, R. Janjam, W. Johns, C. Maguire, A. Melo, H. Ni, P. Sheldon, S. Tuo, J. Velkovska, Q. Xu, M. W. Arenton, P. Barria, B. Cox, J. Goodell, R. Hirosky, A. Ledovskoy, H. Li, C. Neu, T. Sinthuprasith, X. Sun, Y. Wang, E. Wolfe, F. Xia, C. Clarke, R. Harr, P. E. Karchin, J. Sturdy, D. A. Belknap, C. Caillol, S. Dasu, L. Dodd, S. Duric, B. Gomber, M. Grothe, M. Herndon, A. Hervé, P. Klabbers, A. Lanaro, A. Levine, K. Long, R. Loveless, I. Ojalvo, T. Perry, G. A. Pierro, G. Polese, T. Ruggles, A. Savin, N. Smith, W. H. Smith, D. Taylor, N. Woods, CMS Collaboration

**Affiliations:** 1 Yerevan Physics Institute, Yerevan, Armenia; 2 Institut für Hochenergiephysik, Vienna, Austria; 3 Institute for Nuclear Problems, Minsk, Belarus; 4 National Centre for Particle and High Energy Physics, Minsk, Belarus; 5 Universiteit Antwerpen, Antwerp, Belgium; 6 Vrije Universiteit Brussel, Brussels, Belgium; 7 Université Libre de Bruxelles, Brussels, Belgium; 8 Ghent University, Ghent, Belgium; 9 Université Catholique de Louvain, Louvain-la-Neuve, Belgium; 10 Université de Mons, Mons, Belgium; 11 Centro Brasileiro de Pesquisas Fisicas, Rio de Janeiro, Brazil; 12 Universidade do Estado do Rio de Janeiro, Rio de Janeiro, Brazil; 13 Universidade Estadual Paulista, Universidade Federal do ABC, São Paulo, Brazil; 14 Institute for Nuclear Research and Nuclear Energy, Sofia, Bulgaria; 15 University of Sofia, Sofia, Bulgaria; 16 Beihang University, Beijing, China; 17 Institute of High Energy Physics, Beijing, China; 18 State Key Laboratory of Nuclear Physics and Technology, Peking University, Beijing, China; 19 Universidad de Los Andes, Bogotá, Colombia; 20 Faculty of Electrical Engineering, Mechanical Engineering and Naval Architecture, University of Split, Split, Croatia; 21 Faculty of Science, University of Split, Split, Croatia; 22 Institute Rudjer Boskovic, Zagreb, Croatia; 23 University of Cyprus, Nicosia, Cyprus; 24 Charles University, Prague, Czech Republic; 25 Universidad San Francisco de Quito, Quito, Ecuador; 26 Academy of Scientific Research and Technology of the Arab Republic of Egypt, Egyptian Network of High Energy Physics, Cairo, Egypt; 27 National Institute of Chemical Physics and Biophysics, Tallinn, Estonia; 28 Department of Physics, University of Helsinki, Helsinki, Finland; 29 Helsinki Institute of Physics, Helsinki, Finland; 30 Lappeenranta University of Technology, Lappeenranta, Finland; 31 IRFU, CEA, Université Paris-Saclay, Gif-sur-Yvette, France; 32 Laboratoire Leprince-Ringuet, Ecole Polytechnique, IN2P3-CNRS, Palaiseau, France; 33 Institut Pluridisciplinaire Hubert Curien, Université de Strasbourg, Université de Haute Alsace Mulhouse, CNRS/IN2P3, Strasbourg, France; 34 Centre de Calcul de l’Institut National de Physique Nucleaire et de Physique des Particules, CNRS/IN2P3, Villeurbanne, France; 35 Université de Lyon, Université Claude Bernard Lyon 1, CNRS-IN2P3, Institut de Physique Nucléaire de Lyon, Villeurbanne, France; 36 Georgian Technical University, Tbilisi, Georgia; 37 Tbilisi State University, Tbilisi, Georgia; 38 RWTH Aachen University, I. Physikalisches Institut, Aachen, Germany; 39 RWTH Aachen University, III. Physikalisches Institut A, Aachen, Germany; 40 RWTH Aachen University, III. Physikalisches Institut B, Aachen, Germany; 41 Deutsches Elektronen-Synchrotron, Hamburg, Germany; 42 University of Hamburg, Hamburg, Germany; 43 Institut für Experimentelle Kernphysik, Karlsruhe, Germany; 44 Institute of Nuclear and Particle Physics (INPP), NCSR Demokritos, Aghia Paraskevi, Greece; 45 National and Kapodistrian University of Athens, Athens, Greece; 46 University of Ioánnina, Ioannina, Greece; 47 MTA-ELTE Lendület CMS Particle and Nuclear Physics Group, Eötvös Loránd University, Budapest, Hungary; 48 Wigner Research Centre for Physics, Budapest, Hungary; 49 Institute of Nuclear Research ATOMKI, Debrecen, Hungary; 50 Institute of Physics, University of Debrecen, Debrecen, Hungary; 51 National Institute of Science Education and Research, Bhubaneswar, India; 52 Panjab University, Chandigarh, India; 53 University of Delhi, Delhi, India; 54 Saha Institute of Nuclear Physics, Kolkata, India; 55 Indian Institute of Technology Madras, Madras, India; 56 Bhabha Atomic Research Centre, Mumbai, India; 57 Tata Institute of Fundamental Research-A, Mumbai, India; 58 Tata Institute of Fundamental Research-B, Mumbai, India; 59 Indian Institute of Science Education and Research (IISER), Pune, India; 60 Institute for Research in Fundamental Sciences (IPM), Tehran, Iran; 61 University College Dublin, Dublin, Ireland; 62 INFN Sezione di Bari, Università di Bari, Politecnico di Bari, Bari, Italy; 63 INFN Sezione di Bologna, Università di Bologna, Bologna, Italy; 64 INFN Sezione di Catania, Università di Catania, Catania, Italy; 65 INFN Sezione di Firenze, Università di Firenze, Florence, Italy; 66 INFN Laboratori Nazionali di Frascati, Frascati, Italy; 67 INFN Sezione di Genova, Università di Genova, Genoa, Italy; 68 INFN Sezione di Milano-Bicocca, Università di Milano-Bicocca, Milan, Italy; 69 INFN Sezione di Napoli, Università di Napoli ‘Federico II’ Naples, Italy, Università della Basilicata, Potenza, Italy, Università G. Marconi, Rome, Italy; 70 INFN Sezione di Padova, Università di Padova, Padua, Italy, Università di Trento, Trento, Italy; 71 INFN Sezione di Pavia, Università di Pavia, Pavia, Italy; 72 INFN Sezione di Perugia, Università di Perugia, Perugia, Italy; 73 INFN Sezione di Pisa, Università di Pisa, Scuola Normale Superiore di Pisa, Pisa, Italy; 74 INFN Sezione di Roma, Università di Roma, Rome, Italy; 75 INFN Sezione di Torino, Università di Torino, Turin, Italy, Università del Piemonte Orientale, Novara, Italy; 76 INFN Sezione di Trieste, Università di Trieste, Trieste, Italy; 77 Kyungpook National University, Daegu, Korea; 78 Chonbuk National University, Jeonju, Korea; 79 Chonnam National University, Institute for Universe and Elementary Particles, Kwangju, Korea; 80 Hanyang University, Seoul, Korea; 81 Korea University, Seoul, Korea; 82 Seoul National University, Seoul, Korea; 83 University of Seoul, Seoul, Korea; 84 Sungkyunkwan University, Suwon, Korea; 85 Vilnius University, Vilnius, Lithuania; 86 National Centre for Particle Physics, Universiti Malaya, Kuala Lumpur, Malaysia; 87 Centro de Investigacion y de Estudios Avanzados del IPN, Mexico City, Mexico; 88 Universidad Iberoamericana, Mexico City, Mexico; 89 Benemerita Universidad Autonoma de Puebla, Puebla, Mexico; 90 Universidad Autónoma de San Luis Potosí, San Luis Potosí, Mexico; 91 University of Auckland, Auckland, New Zealand; 92 University of Canterbury, Christchurch, New Zealand; 93 National Centre for Physics, Quaid-I-Azam University, Islamabad, Pakistan; 94 National Centre for Nuclear Research, Swierk, Poland; 95 Institute of Experimental Physics, Faculty of Physics, University of Warsaw, Warsaw, Poland; 96 Laboratório de Instrumentação e Física Experimental de Partículas, Lisbon, Portugal; 97 Joint Institute for Nuclear Research, Dubna, Russia; 98 Petersburg Nuclear Physics Institute, Gatchina (St. Petersburg), Russia; 99 Institute for Nuclear Research, Moscow, Russia; 100 Institute for Theoretical and Experimental Physics, Moscow, Russia; 101 Moscow Institute of Physics and Technology, Moscow, Russia; 102 National Research Nuclear University ’Moscow Engineering Physics Institute’ (MEPhI), Moscow, Russia; 103 P.N. Lebedev Physical Institute, Moscow, Russia; 104 Skobeltsyn Institute of Nuclear Physics, Lomonosov Moscow State University, Moscow, Russia; 105 Novosibirsk State University (NSU), Novosibirsk, Russia; 106 State Research Center of Russian Federation, Institute for High Energy Physics, Protvino, Russia; 107 Faculty of Physics and Vinca Institute of Nuclear Sciences, University of Belgrade, Belgrade, Serbia; 108 Centro de Investigaciones Energéticas Medioambientales y Tecnológicas (CIEMAT), Madrid, Spain; 109 Universidad Autónoma de Madrid, Madrid, Spain; 110 Universidad de Oviedo, Oviedo, Spain; 111 Instituto de Física de Cantabria (IFCA), CSIC-Universidad de Cantabria, Santander, Spain; 112 CERN, European Organization for Nuclear Research, Geneva, Switzerland; 113 Paul Scherrer Institut, Villigen, Switzerland; 114 Institute for Particle Physics, ETH Zurich, Zurich, Switzerland; 115 Universität Zürich, Zurich, Switzerland; 116 National Central University, Chung-Li, Taiwan; 117 National Taiwan University (NTU), Taipei, Taiwan; 118 Department of Physics, Faculty of Science, Chulalongkorn University, Bangkok, Thailand; 119 Physics Department, Science and Art Faculty, Cukurova University, Adana, Turkey; 120 Physics Department, Middle East Technical University, Ankara, Turkey; 121 Bogazici University, Istanbul, Turkey; 122 Istanbul Technical University, Istanbul, Turkey; 123 Institute for Scintillation Materials of National Academy of Science of Ukraine, Kharkov, Ukraine; 124 National Scientific Center, Kharkov Institute of Physics and Technology, Kharkov, Ukraine; 125 University of Bristol, Bristol, UK; 126 Rutherford Appleton Laboratory, Didcot, UK; 127 Imperial College, London, UK; 128 Brunel University, Uxbridge, UK; 129 Baylor University, Waco, USA; 130 The University of Alabama, Tuscaloosa, USA; 131 Boston University, Boston, USA; 132 Brown University, Providence, USA; 133 University of California, Davis, Davis, USA; 134 University of California, Los Angeles, USA; 135 University of California, Riverside, Riverside, USA; 136 University of California, San Diego, La Jolla, USA; 137 Department of Physics, University of California, Santa Barbara, Santa Barbara, USA; 138 California Institute of Technology, Pasadena, USA; 139 Carnegie Mellon University, Pittsburgh, USA; 140 University of Colorado Boulder, Boulder, USA; 141 Cornell University, Ithaca, USA; 142 Fairfield University, Fairfield, USA; 143 Fermi National Accelerator Laboratory, Batavia, USA; 144 University of Florida, Gainesville, USA; 145 Florida International University, Miami, USA; 146 Florida State University, Tallahassee, USA; 147 Florida Institute of Technology, Melbourne, USA; 148 University of Illinois at Chicago (UIC), Chicago, USA; 149 The University of Iowa, Iowa City, USA; 150 Johns Hopkins University, Baltimore, USA; 151 The University of Kansas, Lawrence, USA; 152 Kansas State University, Manhattan, USA; 153 Lawrence Livermore National Laboratory, Livermore, USA; 154 University of Maryland, College Park, USA; 155 Massachusetts Institute of Technology, Cambridge, USA; 156 University of Minnesota, Minneapolis, USA; 157 University of Mississippi, Oxford, USA; 158 University of Nebraska-Lincoln, Lincoln, USA; 159 State University of New York at Buffalo, Buffalo, USA; 160 Northeastern University, Boston, USA; 161 Northwestern University, Evanston, USA; 162 University of Notre Dame, Notre Dame, USA; 163 The Ohio State University, Columbus, USA; 164 Princeton University, Princeton, USA; 165 University of Puerto Rico, Mayaguez, USA; 166 Purdue University, West Lafayette, USA; 167 Purdue University Calumet, Hammond, USA; 168 Rice University, Houston, USA; 169 University of Rochester, Rochester, USA; 170 Rutgers,The State University of New Jersey, Piscataway, USA; 171 University of Tennessee, Knoxville, USA; 172 Texas A&M University, College Station, USA; 173 Texas Tech University, Lubbock, USA; 174 Vanderbilt University, Nashville, USA; 175 University of Virginia, Charlottesville, USA; 176 Wayne State University, Detroit, USA; 177 University of Wisconsin-Madison, Madison, WI USA; 178 CERN, 1211 Geneva 23, Switzerland

## Abstract

The cross section of top quark–antiquark pair production in proton–proton collisions at ${\sqrt{s}} = 13\,\text{TeV}$ is measured by the CMS experiment at the LHC, using data corresponding to an integrated luminosity of 2.2$\,\text{fb}^{-1}$. The measurement is performed by analyzing events in which the final state includes one electron, one muon, and two or more jets, at least one of which is identified as originating from hadronization of a b quark. The measured cross section is $815 \pm 9\,\text{(stat)} \pm 38\,\text{(syst)} \pm 19\,\text{(lumi) pb}$, in agreement with the expectation from the standard model.

## Introduction

The measurement of the top quark–antiquark pair ($\mathrm{t}\overline{\mathrm{t}}$) cross section provides a test of the hadroproduction of top quark pairs as predicted by quantum chromodynamics (QCD). At the CERN LHC, measurements have been performed in many different decay channels and at three different proton–proton collision energies [1–24]. Precision measurements of these cross sections allow for a test of their energy dependence as predicted by QCD; they can also place constrains on the parton distribution functions (PDFs) [25]. In combination with some theory, they also provide unambiguous measurements of interesting quantities, such as the top quark pole mass [13, 21], which is difficult to determine by other means. A detailed understanding of the production cross section is also required in searches for evidence of new physics beyond the standard model, as $\mathrm{t}\overline{\mathrm{t}}$ production is often the dominant background process. This is especially important if the signature for the new physics is similar to that of $\mathrm{t}\overline{\mathrm{t}}$ production [13, 26]. This paper presents a measurement of the $\mathrm{t}\overline{\mathrm{t}}$ production cross section ($\sigma _{\mathrm{t}\overline{\mathrm{t}}}$) in the $\mathrm {e}^\pm \mathrm {\mu }^\mp$ decay channel using an event-counting method, based on observed yields. The analysis follows closely [12], and uses the full data set recorded by CMS at 13$\,\text {TeV}$ during 2015, which corresponds to an integrated luminosity of 2.2$\,\text {fb}^{-1}$. This represents a factor of 50 increase in the amount of data over the original analysis and allows for more detailed studies of the experimental and theory uncertainties.

## The CMS detector and Monte Carlo simulation

The CMS detector [27] has a superconducting solenoid in its central region that provides an axial magnetic field of 3.8$\text {\,T}$. The silicon pixel and strip trackers cover $0< \phi <2\pi$ in azimuth and $|\eta |<2.5$ in pseudorapidity. The lead tungstate crystal electromagnetic calorimeter, and the brass and scintillator hadron calorimeter are located inside the solenoid. These are used to identify electrons, photons and jets. Muons are measured in gas-ionization detectors embedded in the steel flux-return yoke outside the solenoid. The detector is nearly hermetic, providing reliable measurement of the momentum imbalance in the plane transverse to the beams. A two-level trigger system selects the most interesting $\mathrm {p}\mathrm {p}$ collisions for offline analysis. A more detailed description of the CMS detector, together with a definition of the coordinate system used and the relevant kinematic variables, can be found in Ref. [27].

Different Monte Carlo (MC) event generators are used to simulate signal and background events. The next-to-leading-order (NLO) powheg  (v2) [28, 29] generator is used for $\mathrm{t}\overline{\mathrm{t}}$ events, with the top quark mass ($m_{\mathrm{t}}$) set to 172.5$\,\text {GeV}$. The NNPDF3.0 NLO [30] PDFs are used. For the reference $\mathrm{t}\overline{\mathrm{t}}$ sample, the events are interfaced with pythia  (v8.205) [31, 32] with the CUETP8M1 tune [33, 34] to simulate parton showering, hadronization, and the underlying event. Additional samples are produced by showering the events in the reference sample with herwig++  (v2.7.1) [35] or by generating events using mg5_amc@nlo  (v5_2.2.2) [36] interfaced with madspin  [37] to account for spin correlations in the decays of the top quarks, and using pythia for parton showering and hadronization.

The mg5_amc@nlo generator is also used to simulate $\mathrm {W}$+jets events and Drell–Yan (DY) quark–antiquark annihilation into lepton-antilepton pairs through a virtual photon or a Z boson exchange; for these backgrounds the event yields are estimated from data. Single top quark events are simulated using powheg  (v1) [38, 39] and pythia, and the event yields are normalized to the approximate next-to-next-to-leading order (NNLO) cross sections from Ref. [40]. The diagram removal approach [41] is used to handle the interference between the $\mathrm{t}\overline{\mathrm{t}}$ and tW final states starting at NLO. The contributions from $\mathrm {W}$ $\mathrm {W}$, $\mathrm {W}$ $\mathrm{Z}$, and $\mathrm{Z}$ $\mathrm{Z}$ (referred to as “VV”) processes are simulated with pythia, and the event rates are normalized to the NLO cross sections from Ref. [42]. Other contributions from $\mathrm {W}$ and $\mathrm{Z}$ boson production in association with $\mathrm{t}\overline{\mathrm{t}}$ events (referred to as “$\mathrm{t}\overline{\mathrm{t}}$V”) are simulated using mg5_amc@nlo  and pythia. The simulated samples include additional interactions per bunch crossing (pileup), with the distribution matching that observed in data, with an average of about 11 collisions per bunch crossing.

The SM prediction for $\sigma _{\mathrm{t}\overline{\mathrm{t}}}$ at 13$\,\text {TeV}$ is $832^{+20}_{-29}\,\text {(scales)}\pm 35\,$(PDF$+\alpha _s)\text {\,pb}$ for $m_{\mathrm{t}}=172.5\,\text {GeV}$, as calculated with the Top++ program [43] at NNLO in perturbative QCD, including soft-gluon resummation at next-to-next-to-leading-log order [44]. The first uncertainty reflects uncertainties in the factorization ($\mu _\mathrm {F}$) and renormalization ($\mu _\mathrm {R}$) scales. The second one is associated with possible choices of PDFs and the value of the strong coupling constant, following the PDF4LHC prescriptions [45, 46], using the MSTW2008 68% confidence level NNLO [47, 48], CT10 NNLO [49, 50], and NNPDF2.3 5f FFN [51] PDF sets. The expected event yields for signal in all figures and tables are normalized to this cross section.

## Event selection

In the SM, top quarks in $\mathrm {p}\mathrm {p}$ collisions are mostly produced as $\mathrm{t}\overline{\mathrm{t}}$ pairs, where each top quark decays predominantly to a $\mathrm {W}$ boson and a bottom quark. In $\mathrm{t}\overline{\mathrm{t}}$ events where both $\mathrm {W}$ bosons decay leptonically, the final state contains two leptons of opposite electric charge and at least two jets coming from the hadronization of the bottom quarks.

At the trigger level, a combination of the single lepton and dilepton triggers is used. Events are required to contain either one electron with transverse momentum $p_{\mathrm {T}} > 12\,\text {GeV}$ and one muon with $p_{\mathrm {T}} > 17\,\text {GeV}$ or one electron with $p_{\mathrm {T}} > 17\,\text {GeV}$ and one muon with $p_{\mathrm {T}} > 8\,\text {GeV}$. In addition, single-lepton triggers with one electron (muon) with $p_{\mathrm {T}} > 23\,\text {GeV}$ (20) are used in order to increase the efficiency. The efficiency for the combination of the single lepton and dilepton triggers is measured in data using triggers based on $p_{\mathrm {T}}$ imbalance in the event. The trigger efficiency is measured to be $0.99 \pm 0.01$ (combined statistical and systematic uncertainties) when the selection on the leptons described below is applied. The trigger in simulation is corrected using a multiplicative data-to-simulation scale factor (SF), given by the trigger efficiency measured in data with independent monitoring triggers.

The particle-flow (PF) event algorithm [52, 53] reconstructs and identifies each individual particle with an optimized combination of information from the various elements of the CMS detector. Selected dilepton events are required to contain one isolated electron [54] and one isolated muon [55] with opposite electric charge and $p_{\mathrm {T}} > 20\,\text {GeV}$ and $|\eta | < 2.4$. Isolation requirements are based on the ratio of the scalar sum of the transverse momenta of all PF candidates, reconstructed inside a cone centered on the lepton, excluding the contribution from the lepton candidate. This isolation variable is required to be smaller than 7% (15%) of the electron (muon) $p_{\mathrm {T}}$.

In events with more than one pair of leptons passing the selection, the two opposite-sign different-flavour leptons with the largest $p_{\mathrm {T}}$ are selected for further study. Events with $\mathrm {W}$ bosons decaying into $\mathrm {\tau }$ leptons contribute to the measurement only if the $\mathrm {\tau }$ leptons decay into electrons or muons that satisfy the selection requirements.

The efficiency of the lepton selection is measured using a “tag-and-probe” [56] method in a sample of same-flavour dilepton events, which is enriched in Z boson candidates. The measured $p_{\mathrm {T}}$- and $\eta$-dependent values for the combined identification and isolation efficiencies average to about 80% for electrons and 90% for muons. To account for the difference in efficiencies determined using data and simulation, the event yield in simulation is corrected using $p_{\mathrm {T}}$- and $\eta$-dependent SFs based on a comparison of lepton selection efficiencies in data and simulation. These have an average of 0.99 for electrons and 0.98 for muons.

In order to suppress backgrounds from DY production of $\tau$ lepton pairs with low invariant dilepton mass, $\mathrm{t}\overline{\mathrm{t}}$ candidate events are further required to have a dilepton pair of invariant mass $m_{\mathrm {e}\mu } > 20\,\text {GeV}$.

Jets are reconstructed from the PF particle candidates using the anti-$k_t$ clustering algorithm [57, 58] with a distance parameter of 0.4. The jet momentum is determined from the vectorial sum of all particle momenta in the jet, and is found from simulation to be within 5 to 10% of the true momentum over the whole $p_{\mathrm {T}}$ spectrum and detector acceptance. An offset correction is applied to jet energies to take into account the contribution from additional proton–proton interactions within the same or nearby bunch crossings. Jet energy corrections are derived from simulation, confirmed with in situ measurements of the energy balance in dijet and photon + jet events, and are applied as a function of the jet $p_{\mathrm {T}}$ and $\eta$ [59] to both data and simulated events. The $\mathrm{t}\overline{\mathrm{t}}$ candidate events are required to have at least two reconstructed jets with $p_{\mathrm {T}} > 30\,\text {GeV}$ and $|\eta |< 2.4$.

Since $\mathrm{t}\overline{\mathrm{t}}$ events decay into final states containing a bottom quark–antiquark pair, requiring the presence of jets identified as originating from $\mathrm{b}$ quarks (“$\mathrm{b}$ jets”) reduces backgrounds from DY and $\mathrm {W}$+jets production. Jets are identified as $\mathrm{b}$ jets using the combined secondary vertex algorithm  [60, 61], with an operating point which yields an identification efficiency of 67% and a misidentification (mistag) probability of about 1% and 15% [61] for light-flavour jets ($\mathrm{u}$, $\mathrm{d}$, $\mathrm{s}$, and gluons) and $\mathrm{c}$ jets, respectively. The selection requires the presence of at least one $\mathrm{b}$ jet in the event.

## Background determination

Background events arise primarily from single top quark, DY, and VV events in which at least two prompt leptons are produced by Z or $\mathrm {W}$ boson decays. The single top quark and VV contributions are estimated from simulation.

The DY event yield is estimated from data using the “$R_\text {out/in}$” method [1, 2, 6], where events with same-flavour leptons are used to normalize the yield of $\mathrm {e}^\pm \mathrm {\mu }^\mp$ pairs from DY production of $\tau$ lepton pairs. A data-to-simulation normalization factor is estimated from the number of events in data within a 15$\,\text {GeV}$ window around the Z boson mass and extrapolated to the number of events outside the Z mass window with corrections applied using control regions enriched in DY events in data. The SF is found to be $0.95 \pm 0.05$ (statistical uncertainty) after applying the final event selection.

Other background sources, such as $\mathrm{t}\overline{\mathrm{t}}$ or $\mathrm {W}$+jets events in the lepton+jets final state, can contaminate the signal sample if a jet is incorrectly reconstructed as a lepton, or the lepton is incorrectly identified as being isolated. This is more important for electrons. For muons, the dominant contribution comes from the semileptonic decay of bottom or charm quarks. These events are grouped into the nonprompt leptons category (“non-$\mathrm {W}$/Z leptons”) since prompt leptons are defined as originating from decays of $\mathrm {W}$ or Z boson, together with contributions that can arise, for example, from decays of mesons or photon conversions.

The contribution of non-$\mathrm {W}$/Z lepton events is estimated from a control region of same-sign (SS) events and propagated in the opposite-sign (OS) signal region. The SS control region is defined using the same criteria as the nominal signal region, except for requiring $\mathrm {e}$ $\mathrm {\mu }$ pairs with the same electric charge. The SS dilepton events are predominantly events containing misidentified leptons. Other SM processes produce prompt SS or charge-misidentified dilepton events with significantly smaller rates; these are estimated using simulation and subtracted from the observed number of events in data.

The scaling from the SS control region in data to the signal region is performed through the ratio of the numbers of OS to SS events with misidentified leptons in simulation. This ratio is calculated using simulated $\mathrm{t}\overline{\mathrm{t}}$ and $\mathrm {W}$+jets samples, which are rich in nonprompt dilepton events, and is measured to be $1.4 \pm 0.1\,\text {(stat)}$. In data, 152 SS events are observed, with a contribution of $79.8\pm 1.9\,\text {(stat)}$ prompt lepton SS events as evaluated from simulation. In total $104 \pm 8\,\text {(stat + syst)}$ events with misidentified leptons contaminating the signal region are predicted. This agrees within the uncertainties with predictions from the simulation.

**Fig. 1:**
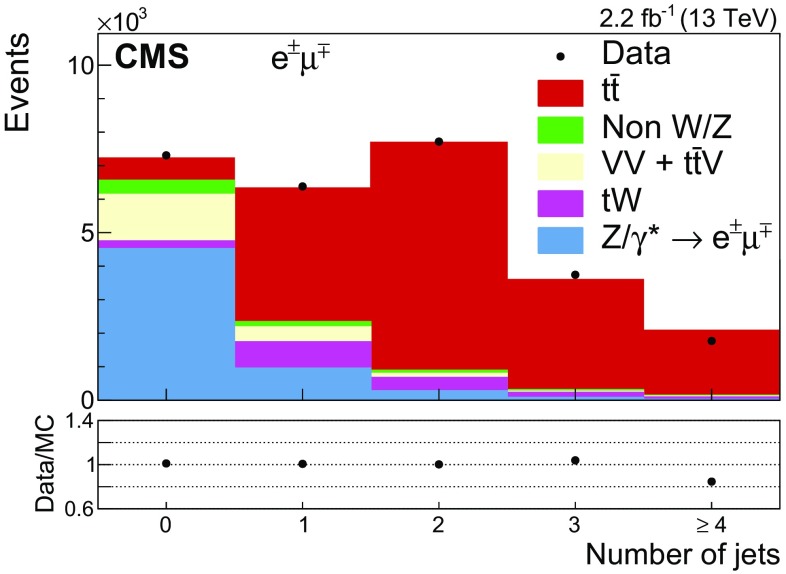
Distribution of the jet multiplicity in events passing the dilepton selection criteria. The expected distributions for $\mathrm{t}\overline{\mathrm{t}}$ signal and individual backgrounds are shown after corrections based on control regions in data are applied; the last bin contains the overflow events. The ratio of data to the sum of the expected yields is given at the *bottom* of the figure. The *error bars*, which are within the size of the points, indicate the statistical uncertainties

**Fig. 2:**
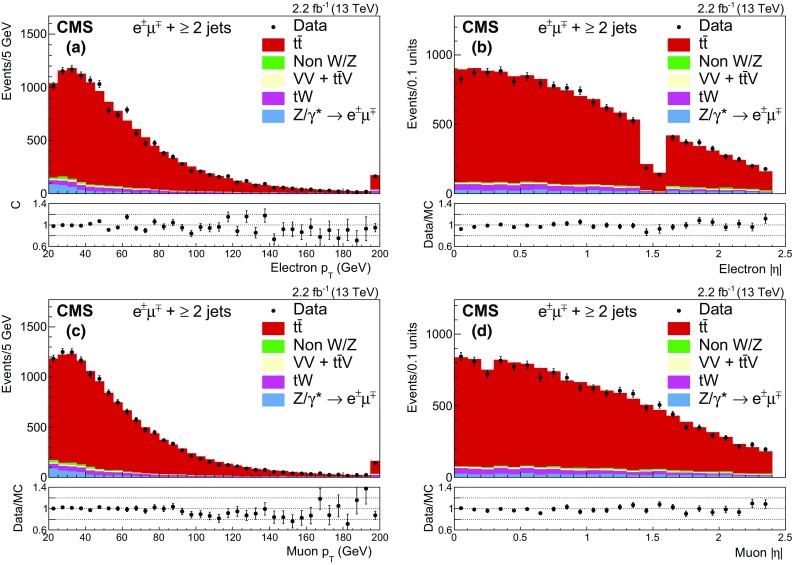
The distributions of **a** $p_{\mathrm {T}}$ and **b** $|\eta |$ of the electron, and **c** $p_{\mathrm {T}}$ and **d** $|\eta |$ of the muon after the selection of jets and before the $\mathrm{b}$ jet requirement. The expected distributions for $\mathrm{t}\overline{\mathrm{t}}$ signal and individual backgrounds are shown after corrections based on control regions in data are applied; for the *left plots* (**a**, **c**) the last bin contains the overflow events. The ratios of data to the sum of the expected yields are given at the *bottom of each panel*. The *error bars* indicate the statistical uncertainties

**Fig. 3:**
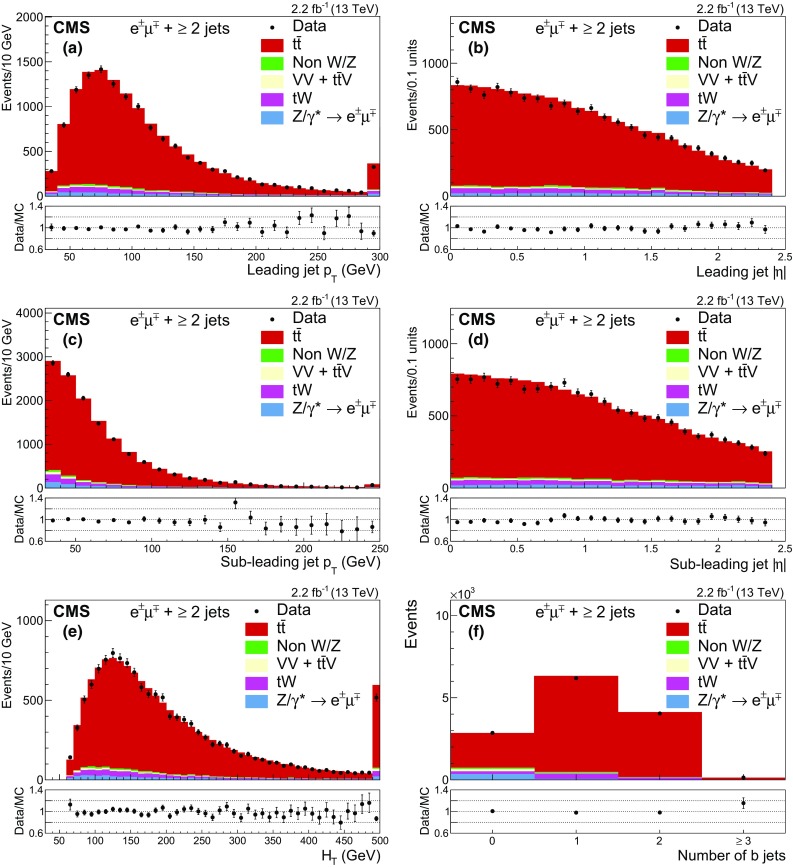
The distributions of **a** $p_{\mathrm {T}}$ and **b** $|\eta |$ for the leading jet, **c** $p_{\mathrm {T}}$ and **d** $|\eta |$ for the sub-leading jet, **e** $H_{\mathrm {T}}$, and **f** b jet multiplicity after the jets selection and before the $\mathrm{b}$ jet requirement. The expected distributions for $\mathrm{t}\overline{\mathrm{t}}$ signal and individual backgrounds are shown after corrections based on control regions in data are applied; in each plot the last bin contains the overflow events. The ratios of data to the sum of the expected yields are given at the *bottom of each panel*. The *error bars* indicate the statistical uncertainties

Figure 1 shows the multiplicity of jets for events passing the dilepton criteria. The MC simulation does not describe well the data for events with $\ge$4 jets, the region in which parton shower effects are expected to dominate the prediction. After requiring at least two jets, Fig. 2 shows the $p_{\mathrm {T}}$ and $|\eta |$ distributions of the selected leptons, and Fig. 3 shows the $p_{\mathrm {T}}$ (a, c) and $|\eta |$ (b, d) distributions of the two most energetic jets; Fig. 3(e) shows the scalar sum of the transverse momenta of all jets ($H_\mathrm {T}$) and Fig. 3(f) the b jet multiplicity. Good agreement between data and the predictions for signal and background is observed.

## Sources of systematic uncertainty

Table 1 summarizes the statistical uncertainty and the different sources of systematic uncertainties in the measured $\mathrm{t}\overline{\mathrm{t}}$ production cross section.

**Table 1:** Summary of the individual contributions to the uncertainty in the $\sigma _{{\mathrm{t}}\overline{\mathrm{t}}}$ measurement. The first and second uncertainty corresponds to the total and relative component, respectively. The total uncertainty in the result, calculated as the quadratic sum of the individual components, is also given

Source	$\Delta \sigma _{{\mathrm{t}}\overline{\mathrm{t}}}$ (pb)	$\Delta \sigma _{\mathrm{t}\overline{\mathrm{t}}} / \sigma _{{\mathrm{t}}\overline{\mathrm{t}}}$ (%)
*Experimental*		
Trigger efficiencies	9.9	1.2
Lepton efficiencies	18.9	2.3
Lepton energy scale	<1	$\le$0.1
Jet energy scale	17.4	2.1
Jet energy resolution	0.8	0.1
b tagging	11.0	1.3
Mistagging	<1	$\le$0.1
Pileup	1.5	0.2
*Modeling*		
$\mu _{\mathrm{F}}$ and $\mu _{\mathrm{R}}$ scales	<1	$\le$0.1
${\mathrm{t}}\overline{\mathrm{t}}$ NLO generator	17.3	2.1
${\mathrm{t}}\overline{\mathrm{t}}$ hadronization	6.0	0.7
Parton shower scale	6.5	0.8
PDF	4.9	0.6
*Background*		
Single top quark	11.8	1.5
VV	<1	$\le$0.1
Drell–Yan	<1	$\le$0.1
Non-${\mathrm{W}}$/Z leptons	2.6	0.3
${\mathrm{t}}\overline{\mathrm{t}}$V	<1	$\le$0.1
Total systematic (no integrated luminosity)	37.8	4.6
Integrated luminosity	18.8	2.3
Statistical	8.5	1.0
Total	43.0	5.3

The uncertainty in the trigger efficiency SF applied to simulation to correct for differences with respect to data is 1.1%. The uncertainty in the SF applied to correct the electron (muon) identification efficiency is found to be about 1.8% (1.5%), with some dependence on the lepton $p_{\mathrm {T}}$ and $\eta$.

The modeling of lepton energy scales was studied using $\mathrm{Z} \rightarrow {\mathrm {e}\mathrm {e}} / \mu \mu$ events in data and simulation, resulting in an uncertainty for the electron (muon) energy scale of 1.0 (0.5)%. These values are used to obtain the effect on the signal acceptance, which is taken as a systematic uncertainty.

The impact of uncertainties in jet energy scale (JES) and jet energy resolution (JER) is estimated from the change observed in the number of simulated $\mathrm{t}\overline{\mathrm{t}}$ events selected after changing the jet momenta within the JES uncertainties, and for JER by an $|\eta |$-dependent variation of the JER scale factors within their uncertainties.

The uncertainties resulting from the $\mathrm{b}$ tagging efficiency and misidentification rate are determined by varying the $\mathrm{b}$ tagging SF of the $\mathrm{b}$ jets and the light-flavour jets, respectively. These uncertainties depend on the $p_{\mathrm {T}}$ and $\eta$ of the jet and amount to approximately 2% for $\mathrm{b}$ jets and 10% for mistagged jets [61] in $\mathrm{t}\overline{\mathrm{t}}$ signal events. They are propagated to the $\mathrm{t}\overline{\mathrm{t}}$ selection efficiency using simulated events.

The uncertainty assigned to the number of pileup events in simulation is obtained by changing the inelastic proton–proton cross section, which is used to estimate the pileup in data, by ±5% [62].

The systematic uncertainty related to the missing higher-order diagrams in powheg is estimated as follows: the uncertainty in the signal acceptance is determined by changing the $\mu _\mathrm {F}$ and $\mu _\mathrm {R}$ scales in powheg independently up and down by a factor of two, with the uncertainty taken as the maximum observed difference.

The predictions of the NLO generators powheg and mg5_amc@nlo for $\mathrm{t}\overline{\mathrm{t}}$ production are compared, where both use pythia for hadronization, fragmentation, and additional radiation description. The difference in the signal acceptance between the two is taken as an uncertainty.

The uncertainty arising from the hadronization model mainly affects the JES and the fragmentation of $\mathrm{b}$ quark jets. The uncertainty in the JES already contains a contribution from the uncertainty in the hadronization. In addition, we determine a related uncertainty by comparing samples of events generated with powheg, where the hadronization is modeled with pythia or herwig++. In what follows we refer to this difference as the hadronization uncertainty.

The impact of the choice of the parton shower scale is studied by changing the scale of the parton shower (initial and final state radiation) by a factor of 2 and 1/2 from its default value. The maximum variation with respect to the central value of the signal acceptance at particle level [63] for the fiducial volume of the analysis is taken as an uncertainty.

The uncertainty from the choice of PDF is determined by reweighting the sample of simulated $\mathrm{t}\overline{\mathrm{t}}$ events according to the NNPDF3.0 PDF sets [30]. The root-mean-square of the distribution is taken as an uncertainty.

Based on recent measurements of the production cross section for single top quark [64–66] and VV [67–74] we use an uncertainty of 30% for these background processes. For DY production, an uncertainty of 15%, that covers the difference of the SF at different levels of the selection, is assumed. A 30% systematic uncertainty is estimated for the non-$\mathrm {W}$/Z lepton background derived from the uncertainty in the ratio of the numbers of OS to SS events with misidentified leptons in the MC simulation.

The uncertainty in the integrated luminosity is 2.3% [75].

## Results

The $\mathrm{t}\overline{\mathrm{t}}$ production cross section is measured by counting events and applying the expression

$$\begin{aligned} \sigma _{\mathrm{t}\overline{\mathrm{t}}} = \frac{N - N_\mathrm {B}}{\mathcal {A} \, {\mathcal {L}}}, \end{aligned}$$

where *N* is the total number of dilepton events observed in data, $N_\mathrm {B}$ is the number of estimated background events, $\mathcal {A}$ is the product of the mean acceptance, the selection efficiency, and the branching fraction into the $\mathrm {e}^\pm \mathrm {\mu }^\mp$ final state, and $\mathcal {L}$ is the integrated luminosity.

Table 2 shows the total number of events observed in data together with the total number of signal and background events determined from simulation or estimated from data. The value of $\mathcal {A}$, determined from simulation assuming $m_{\mathrm{t}}= 172.5\,\text{GeV}$, is $(0.55\pm 0.03) \%$, including statistical and systematic uncertainties. The measured cross section is

$$\begin{aligned} \sigma _{\mathrm{t}\overline{\mathrm{t}}} = 815 \pm 9\,\text{(stat)} \pm 38\,\text{(syst)} \pm 19\,\text{(lumi) pb}, \end{aligned}$$

for a top quark mass of 172.5$\,\text {GeV}$.

**Table 2:** Number of dilepton events obtained after applying the full selection. The results are given for the individual sources of background, $\mathrm{t}\overline{\mathrm{t}}$ signal with a top quark mass of 172.5$\,\text {GeV}$ and $\sigma _{\mathrm{t}\overline{\mathrm{t}}} = 832^{+40}_{-46}\text {\,pb}$, and data. The uncertainties correspond to the statistical component

Source	Number of $\mathrm {e}^\pm \mathrm {\mu }^\mp$ events
Drell–Yan	46 ± 5 ± 7
Non-$\mathrm {W}$/Z leptons	104 ± 8 ± 31
Single top quark	452 ± 6 ± 141
VV	14 ± 2 ± 5
$\mathrm{t}\overline{\mathrm{t}}$V	30 ± 1 ± 9
Total background	646 ± 11 ± 145
$\mathrm{t}\overline{\mathrm{t}}$ signal	9 921 ± 14 ± 436
Data	10368

As a cross-check, analogous measurements have been performed using independent data samples with same-flavour leptons in the final state. The results obtained in the $\mathrm {e}^+\mathrm {e}^-$ and $\mathrm {\mu ^+}\mathrm {\mu ^-}$ channels are consistent with the result in the $\mathrm {e}^\pm \mathrm {\mu }^\mp$ channel. Given their larger uncertainties, the results are not combined with the main one in the $\mathrm {e}^\pm \mathrm {\mu }^\mp$ channel.

The measured fiducial cross section for $\mathrm{t}\overline{\mathrm{t}}$ production with two leptons (one electron and one muon) in the range $p_{\mathrm {T}} > 20\,\text {GeV}$ and $|\eta | < 2.4$, at least two jets with $p_{\mathrm {T}} > 30\,\text {GeV}$ and $|\eta | < 2.4$, and at least one b jet is $\sigma ^\text {fid}_{\mathrm{t}\overline{\mathrm{t}}} = 12.4 \pm 0.1\,\text {(stat)} \pm 0.5\,\text {(syst)} \pm 0.3\,\text{(lumi) pb}$.

The acceptance has been measured in the range 166.5–178.5$\,\text {GeV}$ and is parameterized as a linear function of $m_{\mathrm{t}}$. The cross section varies by 3.7$\text {\,pb}$ when the top quark mass changes 0.5$\,\text {GeV}$.

## Summary

A measurement of the $\mathrm{t}\overline{\mathrm{t}}$ production cross section in proton–proton collisions at ${\sqrt{s}} =13\,\text {TeV}$ is presented for events containing an oppositely charged electron-muon pair, and two or more jets, of which at least one is tagged as originating from a $\mathrm{b}$ quark. The measurement is performed through an event-counting method based on a data sample corresponding to an integrated luminosity of 2.2$\,\text {fb}^{-1}$. The measured cross section is

$$\begin{aligned} \sigma _{\mathrm{t}\overline{\mathrm{t}}} = 815 \pm 9\,\text {(stat)} \pm 38\,\text {(syst)} \pm 19\,\text{(lumi) pb}, \end{aligned}$$

with a total relative uncertainty of 5.3%. The measurement, that supersedes [12], is consistent with recent measurements from the ATLAS [24] and CMS [12] experiments and with the standard model prediction of $\sigma _{\mathrm{t}\overline{\mathrm{t}}} = 832^{+40}_{-46}\text {\,pb}$ for a top quark mass of 172.5$\,\text {GeV}$.
